# Feasibility of cross-vendor linkage of ophthalmic images with electronic health record data: an analysis from the IRIS Registry^®^

**DOI:** 10.1093/jamiaopen/ooae005

**Published:** 2024-01-25

**Authors:** Michael Mbagwu, Zhongdi Chu, Durga Borkar, Alex Koshta, Nisarg Shah, Aracelis Torres, Hylton Kalvaria, Flora Lum, Theodore Leng

**Affiliations:** Verana Health, San Francisco, CA 94107, United States; Byers Eye Institute at Stanford, Stanford University School of Medicine, Palo Alto, CA 94303, United States; Verana Health, San Francisco, CA 94107, United States; Verana Health, San Francisco, CA 94107, United States; Duke Eye Center, Duke University School of Medicine, Durham, NC 27705, United States; Verana Health, San Francisco, CA 94107, United States; Verana Health, San Francisco, CA 94107, United States; Verana Health, San Francisco, CA 94107, United States; Verana Health, San Francisco, CA 94107, United States; American Academy of Ophthalmology, San Francisco, CA 94109, United States; Byers Eye Institute at Stanford, Stanford University School of Medicine, Palo Alto, CA 94303, United States

**Keywords:** clinicoimaging linkage, DICOM, ophthalmic imaging

## Abstract

**Purpose:**

To link compliant, universal Digital Imaging and Communications in Medicine (DICOM) ophthalmic imaging data at the individual patient level with the American Academy of Ophthalmology IRIS^®^ Registry (Intelligent Research in Sight).

**Design:**

A retrospective study using de-identified EHR registry data.

**Subjects, Participants, Controls:**

IRIS Registry records.

**Materials and Methods:**

DICOM files of several imaging modalities were acquired from two large retina ophthalmology practices. Metadata tags were extracted and harmonized to facilitate linkage to the IRIS Registry using a proprietary, heuristic patient-matching algorithm, adhering to HITRUST guidelines. Linked patients and images were assessed by image type and clinical diagnosis. Reasons for failed linkage were assessed by examining patients' records.

**Main Outcome Measures:**

Success rate of linking clinicoimaging and EHR data at the patient level.

**Results:**

A total of 2 287 839 DICOM files from 54 896 unique patients were available. Of these, 1 937 864 images from 46 196 unique patients were successfully linked to existing patients in the registry. After removing records with abnormal patient names and invalid birthdates, the success linkage rate was 93.3% for images. 88.2% of all patients at the participating practices were linked to at least one image.

**Conclusions and Relevance:**

Using identifiers from DICOM metadata, we created an automated pipeline to connect longitudinal real-world clinical data comprehensively and accurately to various imaging modalities from multiple manufacturers at the patient and visit levels. The process has produced an enriched and multimodal IRIS Registry, bridging the gap between basic research and clinical care by enabling future applications in artificial intelligence algorithmic development requiring large linked clinicoimaging datasets.

Key Points **Question:** Can an accurate methodology be developed to link patient records from disparate sources using encoded metadata from ophthalmic images and patient demographic data from a large clinical registry?

**Findings:** Using identifiers from DICOM metadata, we created an automated pipeline to connect longitudinal real-world patient data comprehensively and accurately to various ophthalmic imaging modalities from multiple manufacturers at the patient and clinic visit levels.

**Meaning:** The ability to employ accurate linkage methodology across different ophthalmic imaging instruments in adherence to DICOM standards will support scalable processes, and improved standards compliance will be important for work such as this in the future.

## Introduction

Ophthalmic diagnostic imaging is indispensable to modern clinical practice. Commonly used modalities include optical coherence tomography (OCT), anterior segment and fundus photography, fundus autofluorescence (FAF), fluorescein angiography (FA), and indocyanine green angiography (ICGA). Additionally, imaging is becoming increasingly important for eye screening examinations performed in non-ophthalmology practices to identify patients at the earliest stages of potentially blinding diseases, such as diabetic retinopathy, and as adjuncts when in-person clinical evaluation is difficult or not possible.^1^ These instruments allow for more accurate diagnosis and monitoring of ophthalmic conditions and have revolutionized disease diagnosis and staging over the past several decades.^2^ Modern clinical decision making is also reliant on multifaceted data sources such as these.^3^^,^^4^

There is an abundance of imaging datasets available in ophthalmology,^5^ and electronic health record (EHR) registries are similarly rapidly growing in number. While there is significant scientific potential in combining these two disparate information sources, prior obstacles in these efforts have been substantial.^5^ For example, there is often little and variable compliance by different device manufactures with the global Digital Imaging and Communications in Medicine (DICOM) standards, resulting in difficulties in metadata harmonization.^6^ Additionally, an accurate and reliable methodology is needed for matching patients between two different data sources. Above all, any methodology needs to be conducted in a highly secure manner to protect private patient information.

The American Academy of Ophthalmology IRIS^®^ Registry (Intelligent Research in Sight) is the nation’s first and world’s largest comprehensive eye disease clinical database with over 70 million unique patients and over 12 000 contributing ophthalmologists and is continually growing. In this study, we retrieved and integrated patient-level DICOM file metadata from Heidelberg Engineering (Heidelberg, Germany) and Optos (Dunfermline, United Kingdom) devices and linked them with corresponding clinical and demographic data in the IRIS Registry. The purpose of this study was to assess the feasibility of creating an accurate methodology for successful linkage of clinical and imaging data through the matching of metadata from images to patient demographic data and to evaluate the performance of this linkage. Accurate linkage across different imaging modalities in adherence with the DICOM standard will support the use of these datasets for research and other secondary uses and has the potential to support development of artificial intelligence (AI) algorithms to help answer important questions about eye diseases and conditions.

## Methods

This was a retrospective cohort study of patients included in the IRIS Registry, the world’s largest ophthalmology registry. The IRIS Registry and its use for research purposes have been described in detail previously.^7^ Data stored within the IRIS Registry are deidentified and compliant with the Health Insurance Portability and Accountability Act. All data and analysis adhered to HITRUST^8^ and Mirador^9^ guidelines for privacy and confidentiality. The Western Institutional Review Board Copernicus Group (WCG) reviewed and approved this project and determined that due to the deidentified, retrospective, registry-based nature of our project, written informed consent was waived for this study.^10^ All research adhered to the tenets of the Declaration of Helsinki.

Imaging data were acquired by two practices with Optos^®^ 200Tx, Optos^®^ P200DTx, and Heidelberg Spectralis^®^ and comprehensively normalized and standardized before linkage with EHR data. A format conversion was conducted first to transform manufacturer-specific native imaging data to the standard DICOM format with a licensed Heidelberg proprietary software (HEYEX 2). All converted DICOM files followed DICOM Supplement 91 and Supplement 110 standards.^11^^,^^12^ After all imaging data were standardized to the DICOM format, their metadata were extracted and harmonized to facilitate linking with EHR data. With different imaging data acquired from various modalities and machines, the first two levels of metadata were extracted and concatenated with the following categories of fields: patient, study, series, and image. With the available identifiers extracted from the metadata, a custom linkage algorithm was developed to link patients in the imaging database with patients in the IRIS Registry using the following identifiers: name, gender, medical record number (MRN), birthdate, and practice-related locations. Four sets of combinations were used to determine linkage as follows: location, name, birthdate, and MRN; location, name, birthdate, and gender; location, name, and MRN; and location, birthdate, and MRN. A pair of linkages was considered successful when any set of combinations was linked. Graph theory matching was also implemented to ensure efficient linking. In addition to imaging data, OCT-related key measurements in encapsulated exported PDF files were also obtained and stored in the DICOM format. For each key measurement DICOM file, a service object pair instance unique identifier was provided to link measurements to OCT scans, which had patient information and were linked to the IRIS Registry.

After successful linkage, de-identification of the imaging data was conducted in the same way as de-identification of the EHR data. DICOM metadata fields representing protected health information (PHI) were identified and masked in accordance with an expert-determined certification of the data model (Supplementary Figure S1).^10^ The tag names corresponding to the masked PHI values were still preserved to ensure conformance to the DICOM standard and message integrity. The patient ID field was replaced by an anonymous identifier which was tokenized by a proprietary algorithm.

To explore the cause of a failed linkage, subsequent analysis was performed to assess the number of patients and images with the following criteria: patient with a number or a special character in their name; patient with an abnormal birthdate, defined as before January 1, 1900, or after June 1, 2020(last date of imaging acquisition); and patient with the phrase “test” as part of their name. Patients who fit multiple criteria were counted only once.

With the successfully linked clinicoimaging dataset, a series of descriptive statistics was generated by each image type and by specific disease diagnosis. Image types were defined by the metadata generated by device manufacturer, including OCT, fundus color photography, FAF, FA, ICGA, infrared reflectance (IR), and blue reflectance (BR). Disease diagnoses were defined by International Classification of Diseases (ICD) codes, including diabetic retinopathy with macular edema (DME), diabetic retinopathy without macular edema, exudative age-related macular degeneration (AMD), non-exudative AMD, geographic atrophy (GA), glaucoma, retinal vascular occlusions, choroidal disorders, and hereditary retinal dystrophy. Specific ICD codes used for each diagnosis are listed in Supplementary Table S1. To avoid confusion and complexity, diagnosis in the clinical structured data was linked to patients in the imaging dataset on the patient level, and then all imaging visits and DICOM images were counted for each patient. Patients with multiple diagnoses were allowed to be counted repeatedly among different diagnoses when applicable. Moreover, the relationship between time and the linkage success rate was analyzed. Patients were grouped by the year of their last imaging visit, and the linkage success rate was calculated for each group.

## Results

In total, we acquired 2 287 839 DICOM images from 250 954 clinical visits of 54 896 patients. Of these, we linked 1 937 864 DICOM images from 221 079 clinical visits of 46 196 patients to the IRIS Registry. These images included OCT, fundus color photography, FAF, FA, ICGA, IR, and BR. The initial linkage success rate of EHR patient records to the IRIS Registry patient records was 84.2%. A total of 84.7% of all images were able to be linked to a patient record in the IRIS Registry. Table 1 describes the patient demographics of linked and unlinked records. Table 2 shows the number of patients, visits, and images as well as follow-up statistics by diagnosis, and the distribution of patients, visits, and images by image type and device manufacture. Most unique patients with images in the cohort had a diagnosis of glaucoma, followed by diabetic retinopathy without, and with, macular edema. Patients with non-exudative age-related macular degeneration had the greatest number of unique clinic visits, followed by exudative age-related macular degeneration and glaucoma. There were comparable follow-up times available for all diagnoses, ranging from 1.5 to 2.6 years. With respect to image modalities, IR represented the most frequently used devices, accounting for 40 121 patients, 204 674 visits, and 515 650 unique images generated. With regard to images by vendor, Heidelberg was the most frequently used to obtain patient images in each category except for color fundus photos (Optos, 222 558). Of note, Heidelberg instruments included in the study were not designed or indicated for color fundus photography. Furthermore, we analyzed the linkage success rate of images related to time, namely the year of patients’ last imaging visit in Table 3. Interestingly, there was an increase in linkage success rate when patient visits from earlier timepoints were compared to those that occurred later (2014-2015: 66.53% to 2020-2021: 94.96%). Table 3 also shows the success rate of linkage by imaging type, with linkage success varying from 79% to 91% depending on the type of imaging. Supplementary Table S2 describes the same information in the unlinked records.

**Table 1.** Patient demographics.

Demographic variable		**Linked, *N* = 46** **196**	Unlinked, *N* = 8700
Age in years, mean (SD)		65.45 (17.0)	68.8 (30.4)
Sex	Female	53.5%	56.5%
	Male	45.9%	43.5%
Ethnicity	Hispanic or Latino	12.7%	NA
	Not Hispanic or Latino	75.4%	NA
	Unknown	11.9%	NA
Race	Asian	5.1%	NA
	White	65.7%	NA
	Black or African American	10.1%	NA
	Other	0.3%	NA
	Unknown	18.9%	NA

Note: Unlinked patients lack ethnicity and race information as this information was not recorded in the clinical structured data.

Abbreviations: NA = not applicable, SD = standard deviation.

**Table 2.** Number of patients, visits, images, and follow-up statistics by diagnosis, and distribution of patients, visits, and images by image type and device manufacture.

By diagnosis	Unique patients	Unique visits	Unique images	Follow-up in years, mean (SD)	
Diabetic retinopathy with macular edema	6496	44 102	416 449	1.8 (2.5)	
Diabetic retinopathy without macular edema	7753	51 191	471 788	1.9 (2.5)	
Exudative age-related macular degeneration	5619	65 385	479 541	2.6 (3.0)	
Non-exudative age-related macular degeneration	9533	69 701	548 633	2.0 (2.7)	
Geographic atrophy	1825	16 127	120 122	2.5 (3.0)	
Glaucoma	9095	63 660	506 146	1.8 (2.6)	
Retinal vascular occlusions	4301	29 767	257 020	1.7 (2.4)	
Choroidal disorders	5 756	32 536	300 982	1.7 (2.5)	
Hereditary retinal dystrophy	1782	7210	78 222	1.5 (2.4)	

**By device**	**Unique patients**	**Unique visits**	**Unique images**	**Images from Heidelberg**	**Images from Optos**

OCT	30 087	186 057	381 350	381,350	NA
FAF	21 745	33 517	97 241	49 274	47 967
IR	40 121	204 674	515 650	467 751	47 899
Color	22 218	33 024	222 558	NA	222 558
FA	19 447	26 893	651 901	401 343	250 558
ICGA	1666	2188	45 413	35 165	10 248
BR	2750	3549	7789	7789	NA

Abbreviations: BR = blue reflectance imaging, FA = fluorescein angiography, FAF = fundus autofluorescence, ICGA = indocyanine green angiography, IR = infrared reflectance imaging, OCT = optical coherence tomography, SD = standard deviation.

**Table 3.** Linkage success rate grouped by the year of patients’ last imaging visit and imaging type.

Last imaging visit	Total image count	Linked image count	Linkage success rate (%)
2020-2021	468 767	445 163	94.96
2019-2020	740 436	676 465	91.36
2018-2019	308 612	274 900	89.07
2017-2018	281 791	223 138	79.19
2016-2017	209 847	135 118	64.39
2015-2016	158 928	103 607	65.19
2014-2015	119 458	79 473	66.53

**Image type**	**Total patient count**	**Linked patient count**	**Linkage success rate (%)**

OCT	35 741	30 087	84.18
FAF	24 433	21 745	89.00
IR	47 168	40 121	85.06
Color	24 717	22 218	89.89
FA	22 130	19 447	87.88
ICGA	1828	1666	91.14
BR	3596	2750	76.47

**Figure 1.**
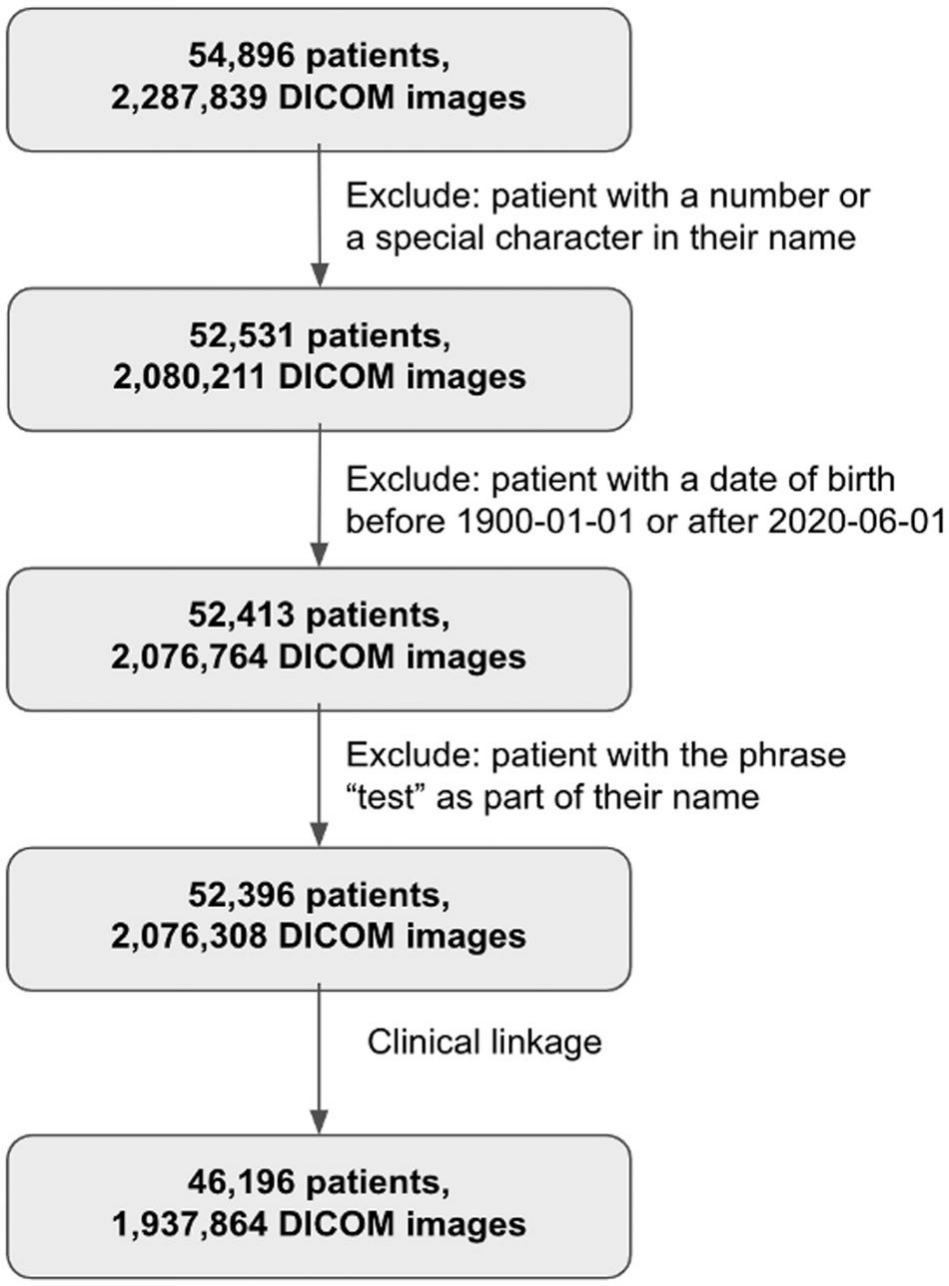
Cohort diagram.

In the unlinked cohort of 8700 patients, an exploration analysis was done to explain the failed linkage. Table 4 lists the number of unlinked images and patients due to abnormal patient name usage and abnormal patient birthdate usage. Abnormal patient name usage includes names with numbers (excluding Roman numerals) and special characters, as well as test patients. Both the patient count and image count were mutually exclusive among rows. After removing these records, our successful linkage rate improved to 93.3% for images and 88.2% for patients, as illustrated in Figure 1.

**Table 4.** Failed linkage due to abnormal patient information.

	Number of patients	Number of images
Names with numbers	2365	207 628
Abnormal date of birth	118	3447
Test patients	17	456

## Discussion

Here we describe a process for leveraging imaging metadata standards to augment an EHR registry with images in a secure and scalable way. Using identifiers from DICOM metadata, we created an automated pipeline to connect longitudinal real-world clinical data comprehensively and accurately to various image modalities from two device manufacturers at the patient and visit levels. Initially, we were able to match 46 196 patients who had a record linked to the IRIS Registry with a success rate of 84.2%. This resulted in 1 937 864 DICOM images matched with a success rate of 84.7%. When imaging metadata with abnormal names or invalid birthdates were excluded from analysis, our overall success rate improved to 88.2% of IRIS Registry-linked patients and 93.3% of all images. The most common reasons for exclusion were patient records that likely represented “test patients” and those with implausible birthdates (before 1900). This suggests that similar methodology may need to be employed in the future to create other large linked clinicoimaging datasets because this same issue is likely to arise.

After successful patient-level linkage, all DICOM images from linked patients were considered linked as well, though it is possible some imaging visits might not have a corresponding clinical visit in the IRIS Registry. This is largely due to the timeframe differences between the IRIS Registry and our DICOM imaging dataset. The IRIS Registry was launched in March 2014,^7^ while some of our images available date back to 2010. An alternative approach would be further linking of imaging visits with clinical visits in the IRIS Registry after successful patient linkage. We did not choose this approach in order to maximize the number of images linked. Because many of the common ocular diseases are age-related diseases and chronic in nature, patients with a diagnosis (ie, AMD) in 2014 would also have a high likelihood of having the same disease from an image obtained on a date prior to the start of the IRIS Registry. Moreover, in ophthalmology, images are commonly used as evidence for diagnosis. Therefore, in the absence of clinical information, clinical diagnosis could also be inferred from images manually or using machine learning algorithms.^13^^,^^14^ In Table 2, the diagnosis was linked on the patient level rather than on the visit level for the same reasons as noted above.

Multi-source data linkage is an important topic in healthcare because it provides several advantages, such as understanding complete patient journeys, cross-validation of diagnoses and procedures, and addressing knowledge gaps in patient care. In ophthalmology, imaging data are often considered the ground truth for diagnosis, and the successful linkage between imaging data and EHR data can work synergistically to create a cross-validated dataset.^15^^,^^16^ With the increased popularity of image-based machine learning algorithms for disease detection, imaging data could also be used to enrich IRIS Registry EHR data, particularly for certain diseases that do not have well-defined ICD codes for diagnosis.

For example, with a linked clinicoimaging dataset, it would be possible to develop a GA diagnosis algorithm based on images because the GA-specific ICD-10 codes were not introduced to clinical usage until 2016. Clinical datasets are also necessary to support the development of AI algorithms because there are areas where the addition of images only may not be useful. Thus far, patient outcome research with the IRIS Registry has largely used clinical outcomes like visual acuity and intraocular pressure. With our linked clinicoimaging dataset, it would be possible to include image-derived outcomes, such as intraretinal fluid volume as well as subretinal fluid volume for DME and exudative AMD patients undergoing anti-vascular endothelial growth factor treatments, as well as GA lesion size for late-stage non-exudative AMD patients to monitor disease progression.

This study included imaging data from two common ophthalmic imaging vendors, due to availability of this equipment at research sites. The success of our algorithm suggests the methodology will be scalable to other ophthalmic imaging vendors as well and will be a topic of future study.

There were some limitations to our study. Our algorithm was able to link between 84.2% and 88.2% of patient records, depending on the methodology used. Since only the four variables present in the DICOM metadata were used to match patient records, it is possible that clinical records may have been inaccurately attributed to imaging metadata due to algorithmic error. Additionally, many images had incomplete information within their metadata (ie, missing names, birthdates, or MRNs). Based on our algorithmic approach that relies on this information, matching would not be possible, making linkages difficult to perform using this methodology.

The ability for EHRs to directly transfer demographic information to ophthalmic imaging equipment is a feature of those systems with the DICOM Modality Worklist feature. While this feature is present with some DICOM-compliant EHR and imaging systems, clinics that still use software versions prior to the implementation do not have this capability. Additionally, many clinics do not have imaging equipment connected directly to EHR systems, making this workflow not possible in these instances. As such, methodology such as ours described may still be necessary to successfully link imaging data to patient records. As indicated in Table 3, the linkage rate increases with time, possibly due to increased compliance to such features and standards. At this time, the IRIS Registry does not contain information on whether EHRs or imaging equipment meets specific DICOM compliance standards, but this will be a topic of future studies.

In summary, we created an accurate and scalable solution for the creation of a linked clinicoimaging dataset from the largest clinical ophthalmology registry, as well as various imaging modalities from two different ophthalmic imaging vendors. This curation process will serve as the basis for an enriched and multimodal IRIS Registry, enabling enhanced research and advanced analytics. As imaging dependence and digital data capture grow, compliance to standards such as DICOM will be critical to advancing data-driven clinical insights to improve quality of care and enable novel ophthalmic drug and device development approaches. Data harmonization between different ophthalmic device manufacturers and modalities in the past has been challenging due to low compliance rates with the DICOM standards, and this remains true today. This presents hurdles to patient image linkage to clinical registries such as the IRIS Registry. Of note, the use of DICOM standards has also been crucial in building similar algorithms in other specialties, such as radiology.^17^ The ability to employ accurate linkage methodology across different imaging instruments in adherence with DICOM standards will support scalable processes, and standards compliance will be important for work such as this in the future.

## Supplementary Material

ooae005_Supplementary_Data

## Data Availability

Available upon request from the corresponding author.

## References

[1] Rajalakshmi R , Prathiba V, Arulmalar S, Usha M. Review of retinal cameras for global coverage of diabetic retinopathy screening. Eye (Lond). 2021;35(1):162-172. doi:10.1038/s41433-020-01262-7 33168977 PMC7852572

[2] Bennett TJ , Barry CJ. Ophthalmic imaging today: an ophthalmic photographer’s viewpoint—a review. Clin Exp Ophthalmol. 2009;37(1):2-13. doi:10.1111/j.1442-9071.2008.01812.x 18947332

[3] Ilginis T , Clarke J, Patel PJ. Ophthalmic imaging. Br Med Bull. 2014;111(1):77-88. doi:10.1093/bmb/ldu022 25139430

[4] Bajwa A , Aman R, Reddy AK. A comprehensive review of diagnostic imaging technologies to evaluate the retina and the optic disk. Int Ophthalmol. 2015;35(5):733-755. doi:10.1007/s10792-015-0087-1 26043677

[5] Khan SM , Liu X, Nath S, et al A global review of publicly available datasets for ophthalmological imaging: barriers to access, usability, and generalisability. Lancet Digit Health. 2021;3(1):e51-e66. doi:10.1016/S2589-7500(20)30240-5 33735069 PMC7618278

[6] Lee AY , Campbell JP, Hwang TS, Lum F, Chew EY, American Academy of Ophthalmology Recommendations for standardization of images in ophthalmology. Ophthalmology. 2021;128(7):969-970. doi:10.1016/j.ophtha.2021.03.003 33832778 PMC8335850

[7] Parke Ii DW , Lum F, Rich WL. The IRIS^®^ Registry: purpose and perspectives. Ophthalmologe. 2017;114(Suppl 1):1-6. doi:10.1007/s00347-016-0265-1 27306823

[8] Health Information Trust Alliance (HITRUST) Common Security Framework (CSF). Accessed January 25, 2022. https://hitrustalliance.net/

[9] Mirador Analytics. Accessed September 26, 2023. https://www.miradoranalytics.com/

[10] Office for Civil Rights (OCR). Guidance Regarding Methods for De-identification of Protected Health Information in Accordance with the Health Insurance Portability and Accountability Act (HIPAA) Privacy Rule. HHS.gov. Published September 7, 2012. Accessed November 4, 2021. https://www.hhs.gov/hipaa/for-professionals/privacy/special-topics/de-identification/index.html

[11] Digital Imaging and Communications in Medicine (DICOM). Supplement 101: Ophthalmic Tomography Image Storage SOP Class. Prepared by DICOM Standards Committee. Published February 2001. Accessed November 4, 2021. https://www.dicomstandard.org/News-dir/ftsup/docs/sups/sup110.pdf

[12] Digital Imaging and Communications in Medicine (DICOM). Supplement 91: Ophthalmic Photography Image SOP Classes. Prepared by DICOM Standards Committee. Published September 2004. Accessed November 4, 2021. https://www.dicomstandard.org/News-dir/ftsup/docs/sups/sup91.pdf

[13] Asaoka R , Murata H, Hirasawa K, et al Using deep learning and transfer learning to accurately diagnose early-onset glaucoma from macular optical coherence tomography images. Am J Ophthalmol. 2019;198:136-145. doi:10.1016/j.ajo.2018.10.007 30316669

[14] Lee CS , Baughman DM, Lee AY. Deep learning is effective for the classification of OCT images of normal versus age-related macular degeneration. Ophthalmol Retina. 2017;1(4):322-327. doi:10.1016/j.oret.2016.12.009 30693348 PMC6347658

[15] Cheng CY , Soh ZD, Majithia S, et al Big data in ophthalmology. Asia Pac J Ophthalmol (Phila). 2020;9(4):291-298. doi:10.1097/APO.0000000000000304 32739936

[16] Bowman S. Impact of electronic health record systems on information integrity: quality and safety implications. Perspect Health Inf Manag. 2013;10(Fall):1c. PMC3797550 24159271

[17] Caffery LJ , Rotemberg V, Weber J, Soyer HP, Malvehy J, Clunie D. The role of DICOM in artificial intelligence for skin disease. Front Med (Lausanne). 2021;7:619787. doi:10.3389/fmed.2020.619787 33644087 PMC7902872

