# Supplementary figures and images for: Feasibility of cross-vendor linkage of ophthalmic images with electronic health record data: an analysis from the IRIS Registry®
Source: JAMIA Open. 2024 Jan 25;7(1):ooae005. doi: 10.1093/jamiaopen/ooae005 (PMC10811449; doi:10.1093/jamiaopen/ooae005)

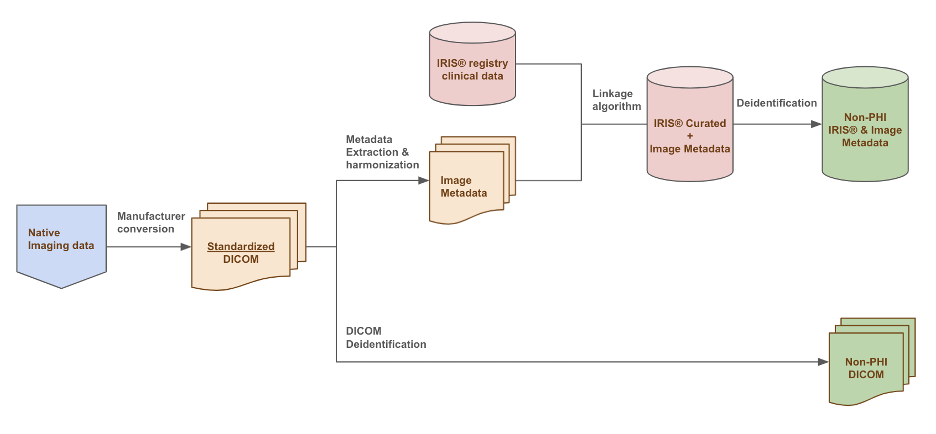

Supplement: ooae005_Supplementary_Data [file ooae005_supplementary_data.zip › Supplementary Figure1.png]
